# Federated learning for privacy-preserving depression detection with multilingual language models in social media posts

**DOI:** 10.1016/j.patter.2024.100990

**Published:** 2024-05-13

**Authors:** Samar Samir Khalil, Noha S. Tawfik, Marco Spruit

**Affiliations:** 1 Computer Engineering Department, Arab Academy for Science, Technology and Maritime Transport, Alexandria, Egypt; 2 Leiden Institute of Advanced Computer Science, Leiden University, 2333 CA Leiden, the Netherlands; 3 Public Health & Primary Care, Leiden University Medical Center, 2333 CA Leiden, the Netherlands

**Keywords:** federated learning, natural language processing, depression, mental health, social media, multilingual

## Abstract

The incidences of mental health illnesses, such as suicidal ideation and depression, are increasing, which highlights the urgent need for early detection methods. There is a growing interest in using natural language processing (NLP) models to analyze textual data from patients, but accessing patients’ data for research purposes can be challenging due to privacy concerns. Federated learning (FL) is a promising approach that can balance the need for centralized learning with data ownership sensitivity. In this study, we examine the effectiveness of FL models in detecting depression by using a simulated multilingual dataset. We analyzed social media posts in five different languages with varying sample sizes. Our findings indicate that FL achieves strong performance in most cases while maintaining clients’ privacy for both independent and non-independent client partitioning.

## Highlights

• Exploring FL’s efficiency in multilingual depression detection task
• Validating results on social media datasets of varying sizes in five languages
• Evaluating FL aggregation algorithms with different client-data partitioning setups

## The bigger picture

Mental illness is a worldwide concern, and the need to develop machine learning models that can adapt to globally diverse linguistic and cultural contexts is crucial. More specifically, natural language processing (NLP) has been utilized to detect, diagnose, and predict various mental disorders. However, the development of such models is constrained by the availability of extensive training data due to data privacy regulations that differ across countries. Federated learning (FL), a machine learning technique that enables collaborative model training, potentially addresses these limitations by allowing data to remain local during training to maintain the confidentiality of patient data.

## Graphical abstract

**Figure:**
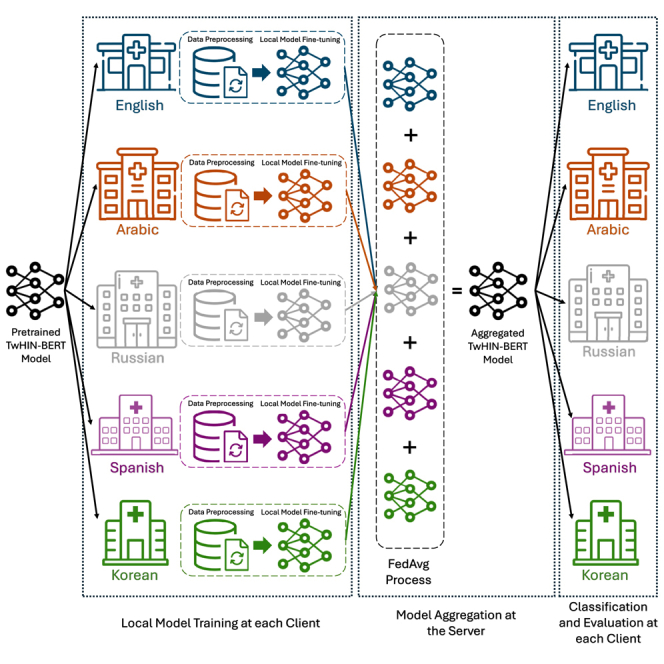

## Teaser

The paper explores federated learning (FL) for detecting depression using patient-authored multilingual textual data. It utilizes five datasets in English, Arabic, Spanish, Russian, and Korean, each varying in size. Findings reveal that FL in IID scenarios matches centralized and local model performance. Multilingual data favor non-IID configurations, prompting exploration of data partitioning strategies to address volume and label distribution differences among clients. Although extreme quantity imbalances reduce model performance, FL emerges as a promising privacy-centric approach for mental health diagnostics.

## Introduction

Rates of mental health conditions are rising, and this urgent issue is driving the development of new treatments and prevention methods. Several factors might be contributing to this increase. These include family instability, the influence of social media, excessive screen time (which some link to electronic screen syndrome), exposure to more divisive news, growing pressure to succeed, and the recent COVID-19 pandemic.^1^ Affecting over 264 million individuals, depression remains the leading cause of global disability according to the World Health Organization.^2^^,^^3^ According to the National Institute of Mental Health, over half of adults aged 18 and above in the US were diagnosed with a mental illness in 2020.^4^ Individuals diagnosed with mental diseases often encounter numerous obstacles in accessing quality healthcare, facing not only systemic barriers but also social stigma and discrimination. As societal awareness of the problems associated with mental illnesses expands, there has been a corresponding rise in efforts to promote mental health education, early intervention strategies, and destigmatization campaigns. This motivated the emergence of various technology-based applications and methods to support mental health prevention, awareness, patient monitoring, and disease identification. The utilization of digital mental health platforms employing machine-learning-driven algorithms to diagnose, treat, and provide care for various psychiatric disorders has proven to be a promising avenue for expanding access to mental healthcare and individualizing treatment plans.

Nearly all algorithms driven by artificial intelligence (AI) struggle with a lack of data in general and the quality of data and labeling in particular. The acquisition and management of health data pose challenges due to privacy considerations and regulatory limitations. Strict data collection, storage, and usage rules are frequently required due to ethical considerations surrounding patient confidentiality and data security. This is particularly pertinent within the mental health field, as patient data are inherently personal and sensitive due to the significant social stigma associated with the patient’s condition. Despite the scarce availability of publicly available datasets, their size often remains limited, restricting the effectiveness of current methodologies.

The concept of federated learning (FL), which involves a collaborative learning technique, was first developed in 2016 by a research team at Google Research.^5^ FL is characterized by a client-server architecture, wherein a centralized model is trained using data distributed over multiple clients (decentralized data). This strategy ensures that the data remain on the client side and are not transmitted to the central server. Although FL was initially developed for a different domain, it has rapidly gained interest in the healthcare and medical sectors because of its potential to address data privacy and governance concerns. This is achieved by enabling collaborative model training without the need for data exchange. The approach proposed ensures a consensus solution without the need to transfer patient data outside the secure boundaries of the healthcare facility where they are stored.^6^

As mentioned above, FL promises that data never leave the client’s side. Instead, a global model learns from data segregated for different clients under the coordination of a centralized server. A key factor is the aggregation algorithm employed; the first and most common one is federated averaging (FedAvg).^5^ In FedAvg, the server computes the aggregated model weights by averaging each client’s model weights based on their share of data (weighted average). In an FL setting, data can be independent and identically distributed (IID) or non-independent and identically distributed (non-IID). IID data distribution occurs when the data are evenly balanced among the clients and the labels are distributed almost uniformly across all clients, ensuring that each class contains a similar number of instances. In practical situations, it is common for data to be non-IID; that is, data at each client cannot be regarded as a subset drawn from the available data at all clients. In other words, the local data available cannot represent the overall data distribution.

FL has been employed in the field of mental health, utilizing several forms of data to investigate its potential in addressing problems such as depression, schizophrenia, suicidal thoughts, bipolar disorder, and other related conditions.^7^^,^^8^^,^^9^^,^^10^ Textual data emerge as the predominant data type in the mental health domain due to their wide availability across various sources. These sources include therapists’ notes, interactions between therapists and patients, counseling sessions, patients’ authored text on social networks or medical forums, and electronic health records. Prior studies on natural language processing (NLP) with FL in the mental health domain were constrained by the premise that the data are locally monolingual or homogeneous or at least have overlapping distributions among clients.^11^^,^^12^^,^^13^^,^^14^ However, only recently has FL been investigated in a multilingual context to identify mental health disorders.^15^ Multilingual FL has been recently explored in different language tasks, as it provides an interesting and natural setting to examine non-IID data, of which different languages are an obvious application.^16^^,^^17^^,^^18^

This research investigates the capabilities of multilingual FL within the mental health domain. We present a proof of concept demonstrating the potential of the FL paradigm in facilitating global collaboration among institutions to develop machine learning models that serve the mental health domain. We focus on depression detection through the analysis of social media posts in five distinct languages.

## Results

This work applied multilingual FL to detect depression using textual data from social media posts in English, Arabic, Russian, Spanish, and Korean. As described in Table 5, all datasets used in our research are sourced from social media platforms. They are balanced and feature post-level annotations, categorizing each post as either “depressed” or “non-depressed.” To evaluate the performance of the FL approach in contrast to the traditional methods in depression detection, we carried out four multicentric setups.

(1) Local models trained locally at each client and divided by language (monolingual).
(2) Centralized, where training combines all available training instances (crosslingual) at a central server without applying any privacy measures.
(3) FL with IID data trained based on FL strategy, where each client has an equal random subset of all data, i.e., divide each dataset across clients. We set the number of clients to 5 to maintain consistency with the non-IID setup of our experiments.
(4) FL with non-IID data, where training data are divided per language, and five clients are each assigned one language.

**Figure 1:**
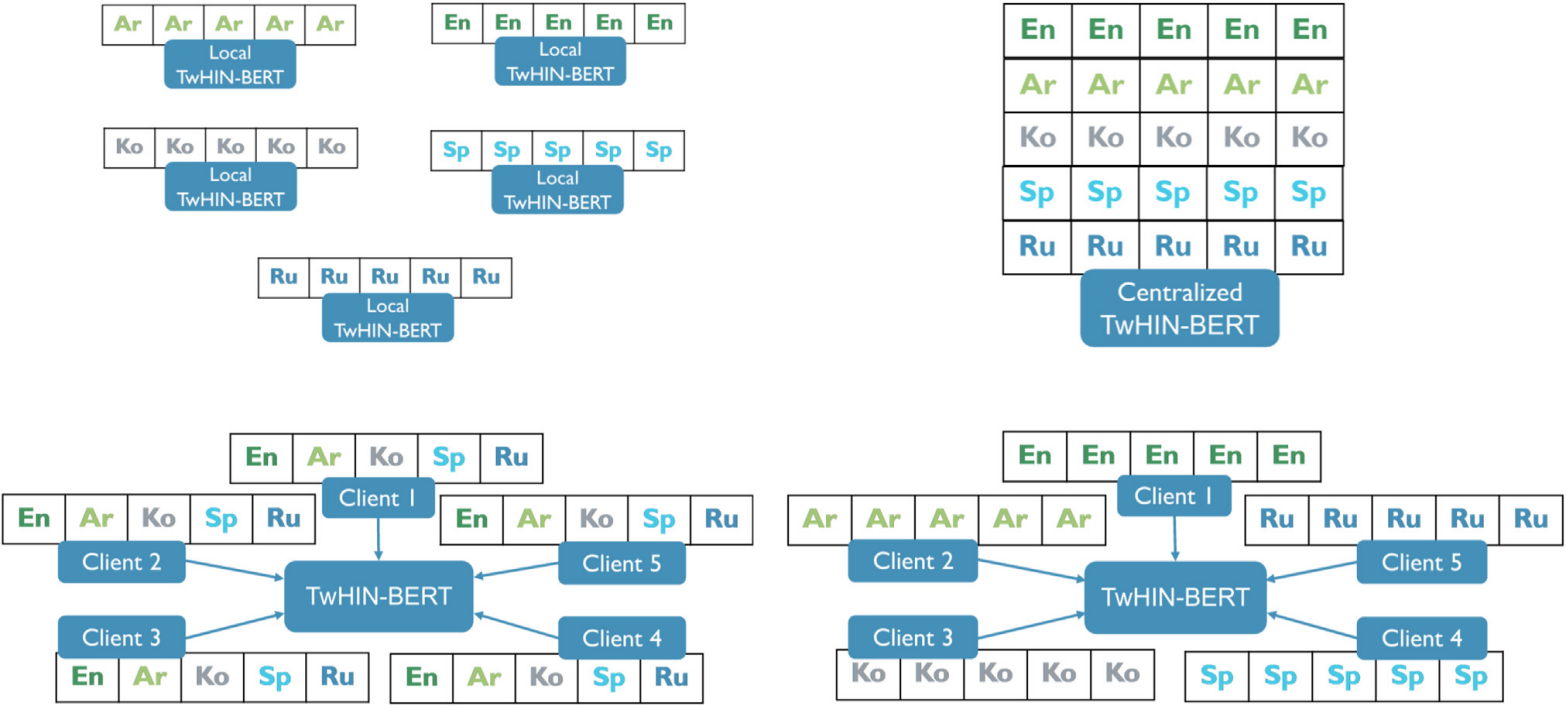
Client partitioning: local (top left), centralized (top right), FL with IID (bottom left) and FL with non-IID (bottom right)

Figure 1 depicts the aforementioned architectural setups. It is worth mentioning that the non-IID scenario corresponds to the nature of multilingual data, in which datasets are typically uneven in size and dispersed based on the geopolitical and legal framework of the region where they are collected. This setup represents five different health facilities in five distinct geographical areas, each speaking a different language.

In each setup, we assessed the performance using area under the receiver operating character curve(AUC) and F1 scores. We conducted a comparison between two multilingual language models (LMs) to determine the most effective model for analyzing multilingual data from social media. The two models are XLM-RoBERTa^19^ (crosslingual language model with robustly optimized bidirectional encoder representations from transformers approach) and TwHIN-BERT^20^ (Twitter heterogeneous information network based on bidirectional encoder representations from transformers).

The premise of independent and equally distributed data is not satisfied in real-world multilingual applications, as it is more probable that each client will hold monolingual data. Hence, owing to the inherent attributes of multilingual data, the datasets are most effectively depicted in scenarios that lack independence and uniform distribution. We performed two further experiments using the non-IID design to investigate this challenge. In some cases in FL, individual clients may possess a locally balanced dataset. However, the collective global dataset of all clients may be imbalanced, or vice versa. Each client might suffer from different imbalances and skews^21^ as follows.

(1) Non-IID with quantity imbalance: where the size of the local dataset varies across clients regardless of the label distribution.
(2) Non-IID with distribution-based label imbalance: where the clients have similar portions of data in terms of size but with different proportions of each label

Moreover, the presence of abundant language data might significantly influence the trained model, resulting in an inadequate representation of languages with limited resources. Four non-IID scenarios are created to investigate the imbalanced nature of multilingual datasets. In addition, we conducted experiments with alternative aggregation algorithms, such as FedProx^22^ (federated optimization in heterogeneous networks) and FedAvgM^23^ (federated averaging with momentum), to assess the impact of the aggregation algorithms on the overall performance. For the FL settings, a total of five clients were included, aligning with the number of languages involved in the study. The FL training was structured over ten rounds. After each round, the centralized model underwent a weight update in the FL setup. We repeated each experiment three times, including the training and evaluation phases. The reported results are the average values and their corresponding standard deviations to confirm the reliability of our findings. The results of the corresponding experiments are shown in Tables 3 and 4; the findings exhibit many considerations and limitations that are shared in the following discussion section.

## Discussion

This study investigates multilingual FL in the mental health domain, precisely the depression detection task from social media posts. Our research covers English, Arabic, Russian, Spanish, and Korean languages, with a total of 24,000 social media posts. State-of-the-art multilingual LMs were employed to analyze input text and classify posts into depression or non-depression classes. We first compared two LMs across four scenarios: local, centralized, FL with IID data, and FL with non-IID data. We aimed to examine the impact of employing an LM that, although pre-trained on a consistent set of languages, underwent additional fine-tuning on X posts to align with the specific nature of our data. As shown in Tables 1 and 2, the TwHIN-BERT model outperforms XLM-RoBERTa with average increases of 1.5%–3% in AUC and 1.53%–2.52% in the F1 score. The rise in performance can be attributed to our original hypothesis that TwHIN-BERT, extensively trained with a social objective, allows the model to effectively represent and predict short, human-generated social media posts, making it more suitable for the prediction task.

**Table 1:** XLM-RoBERTa performance on each language

Setup	Measure	English (%)	Arabic (%)	Russian (%)	Spanish (%)	Korean (%)	Average (%)
Local	AUC	78.7±0.58	96.5±0.26	98.6±0.54	95.0±0.66	96.0±0.50	92.9
Local	F1	79.7±0.10	96.5±0.26	98.6±0.53	95.0±0.64	96.1±0.53	93.2
Centralized	AUC	78.5±1.00	95.7±0.59	98.8±0.06	94.3±0.29	95.8±1.26	92.6
Centralized	F1	81.1±0.57	95.7±0.60	98.8±0.06	94.5±0.24	95.8±1.22	93.2
FL with IID	AUC	80.8±0.58	96.4±0.21	98.9±0.13	95.5±0.50	95.3±0.29	93.4
FL with IID	F1	82.6±0.86	96.4±0.17	98.9±0.13	94.7±0.43	95.4±0.31	93.6
FL with non-IID	AUC	69.2±3.88	95.8±0.31	98.7±0.03	85.8±0.90	76.5±1.50	85.2
FL with non-IID	F1	73.5±2.21	95.9±0.30	98.7±0.03	87.4±0.72	78.7±1.04	86.8

The aggregation algorithm used is FedAvg. The scores are the mean of training with three different runs. ± denotes the standard deviation.

**Table 2:** TwHIN-BERT performance on each language

Setup	Measure	English (%)	Arabic (%)	Russian (%)	Spanish (%)	Korean (%)	Average (%)
Local	AUC	83.3±0.29	97.2±0.37	98.8±0.13	96.7±0.72	98.5±0.50	94.9
Local	F1	84.6±0.48	97.2±0.36	98.8±0.13	96.7±0.69	98.5±0.50	95.1
Centralized	AUC	83.2±0.76	97.1±0.10	98.9±0.16	95.9±0.52	97.7±0.29	94.6
Centralized	F1	84.5±0.89	97.1±0.12	98.9±0.16	95.9±0.61	97.7±0.26	94.8
FL with IID	AUC	83.0±0.87	97.3±0.23	98.8±0.05	97.3±0.25	98.2±0.76	94.9
FL with IID	F1	84.2±0.61	97.3±0.23	98.8±0.05	97.3±0.24	98.2±0.76	95.1
FL with non-IID	AUC	68.3±1.53	97.0±0.58	98.7±0.13	93.3±1.01	84.0±3.12	88.3
FL with non-IID	F1	72.3±1.29	97.0±0.53	98.7±0.14	93.6±0.89	85.0±2.38	89.3

The aggregation algorithm used is FedAvg. The scores are the mean of training with three different runs. ± denotes the standard deviation.

In Table 2, the FL technique demonstrated comparable performance to local and centralized models when applied to Arabic, Russian, and Spanish languages. The Spanish language exhibits a greater variance of 3% than the former two, as it had a smaller share of data. The non-IID performance for English and Korean datasets had a substantial decline, approximately 15% worse than the centralized approach and the FL with IID setup. However, the disparity in performance diminishes in significance as the size of the dataset expands.

We built upon our findings and employed the TwHIN-BERT model in extra non-IID setups. We focused on simulating realistic scenarios where each dataset had a different quantity and label distribution per client, as described in non-IID client partitioning. Results of the corresponding experiments are depicted in Tables 3 and 4. Starting with a balanced non-IID distribution, non-IID:$Q_{b}L_{b}$ denotes a setup where each client has an equal number of samples and a balanced class distribution. This yields a consistent performance across all languages where the difference between FL and centralized results did not exceed 2% in both average AUC and F1. Furthermore, when introducing label distribution imbalance in non-IID:$Q_{b}L_{i}$, the results remain consistent with even less of a performance gap between FL and centralized approaches.

**Table 3:** AUC score for each language in the non-IID setups where data and labels follow Dirichlet distribution

Client partitioning	English (%)	Arabic (%)	Russian (%)	Spanish (%)	Korean (%)	Average (%)
**Non-IID:**$Q_{b}L_{b}$						

C	82.0±0.87	92.3±1.44	99.0±0.00	95.8±1.15	98.5±0.00	93.5
FedAvg	81.0±0.87	89.7±0.76	98.2±1.04	94.5±0.50	95.8±1.53	91.8
FedProx	81.2±1.04	92.5±1.73	98.7±0.58	95.8±0.76	96.3±0.76	92.9
FedAvgM	81.0±0.50	94.8±1.04	98.3±0.76	96.0±0.00	96.2±2.75	93.3

**Non-IID:**$Q_{b}L_{i}$						

C	82.4±0.67	96.6±1.04	98.4±1.09	95.1±1.65	98.1±0.48	94.1
FedAvg	81.0±0.99	95.1±1.68	99.2±0.39	91.7±1.81	96.1±1.59	92.6
FedProx	80.9±0.07	96.3±0.05	98.7±0.45	95.6±1.61	96.4±0.98	93.6
FedAvgM	82.2±0.85	95.2±1.05	98.3±1.36	94.7±3.40	94.9±0.26	93.1

**Non-IID:**$Q_{i}L_{b}$						

C	83.5±2.85	97.4±0.35	98.4±0.68	96.3±0.25	97.4±1.28	94.6
FedAvg	74.4±1.67	97.1±0.24	93.6±1.80	92.0±0.49	85.0±1.48	88.5
FedProx	74.1±1.40	97.2±0.32	94.7±1.58	93.2±0.74	86.8±6.09	89.2
FedAvgM	75.9±3.26	97.4±0.24	93.9±0.98	93.0±1.08	86.8±5.78	89.4

**Non-IID:**$Q_{i}L_{i}$						

C	82.3±2.09	95.8±0.24	99.5±0.63	94.7±0.14	94.5±1.33	93.4
FedAvg	72.2±0.79	95.8±1.53	97.5±1.46	92.4±2.27	82.3±7.31	88.0
FedProx	71.6±2.15	95.3±1.38	97.4±1.52	92.3±3.21	86.0±3.18	88.5
FedAvgM	72.5±1.20	94.0±0.16	96.8±0.54	91.8±2.27	85.1±3.43	88.0

Values are reported in percentages as the mean of 3 training runs using TwHIN-BERT. ± denotes the standard deviation.$Q_{b}L_{b}$, non-IID with balanced quantity and distribution-based label; $Q_{b}L_{i}$, non-IID with distribution-based label imbalance; $Q_{i}L_{b}$, non-IID with quantity imbalance; $Q_{i}L_{i}$, non-IID with quantity and distribution-based label imbalance.

**Table 4:** F1 score for each language in the non-IID setups where data and labels follow Dirichlet distribution

Client partitioning	English (%)	Arabic (%)	Russian (%)	Spanish (%)	Korean (%)	Average (%)
**Non-IID:**$Q_{b}L_{b}$						

C	82.8±0.94	92.9±1.25	99.0±0.01	95.9±1.06	98.5±0.00	93.8
FedAvg	82.2±0.64	90.6±0.63	98.2±1.06	94.7±0.46	96.0±1.46	92.3
FedProx	82.6±1.17	93.0±1.50	98.7±0.59	95.9±0.72	96.4±0.67	93.3
FedAvgM	82.6±0.39	95.1±0.94	98.3±0.78	96.1±0.04	96.4±2.50	93.7

**Non-IID:**$Q_{b}L_{i}$						

C	85.6±0.73	97.4±0.55	96.1±1.02	99.1±0.40	98.0±0.50	95.2
FedAvg	85.2±0.87	96.4±0.71	97.3±0.95	98.6±0.35	94.6±2.30	94.4
FedProx	85.3±0.57	97.2±0.58	96.1±1.05	99.1±0.21	95.1±1.36	94.6
FedAvgM	85.8±0.49	96.6±0.48	96.6±1.73	99.1±0.40	92.6±0.37	94.1

**Non-IID:**$Q_{i}L_{b}$						

C	85.4±1.56	97.4±0.38	98.5±0.64	96.4±0.28	97.4±1.30	95.0
FedAvg	74.9±1.05	97.1±0.22	93.6±1.81	92.2±0.50	85.3±1.52	88.6
FedProx	75.2±1.52	97.2±0.32	94.6±1.60	93.6±0.61	86.5±6.47	89.4
FedAvgM	77.0±4.32	97.4±0.24	93.7±1.22	93.3±1.17	87.2±5.45	89.7

**Non-IID:**$Q_{i}L_{i}$						

C	80.5±2.39	98.4±0.33	99.7±0.37	95.9±0.26	97.6±0.02	94.4
FedAvg	70.6±0.78	98.7±0.17	98.4±0.54	94.6±1.56	93.4±1.05	91.2
FedProx	71.2±0.73	98.6±0.30	98.4±0.62	94.5±2.08	93.4±0.80	91.2
FedAvgM	70.9±1.51	98.4±0.10	97.7±0.52	93.9±1.52	92.5±1.59	90.7

Values are reported in percentages as the mean of 3 training runs using TwHIN-BERT. ± denotes the standard deviation.$Q_{b}L_{b}$, non-IID with balanced quantity and distribution-based label; $Q_{b}L_{i}$, non-IID with distribution-based label imbalance; $Q_{i}L_{b}$, non-IID with quantity imbalance; $Q_{i}L_{i}$, non-IID with quantity and distribution-based label imbalance.

To study the effects of clients’ data heterogeneity, we varied the data size and/or label distribution that reside at each client in non-IID:$Q_{i}L_{b}$ and non-IID:$Q_{i}L_{i}$ following the Dirichlet distribution with β = 0.5. Each client’s data are randomly sampled from the entire dataset without replacement based on the quantity/label ratio of the distribution. In non-IID:$Q_{i}L_{b}$ and non-IID:$Q_{i}L_{i}$, all languages show a gap in the performance for non-IID FL when compared to the Arabic language performance. In the non-IID:$Q_{i}L_{b}$ experiment, the Arabic data amount to almost 60% of the total available data; however, the results are still acceptable for the Russian and Spanish languages. Our overall observation, by looking at the performance average, is that the non-IID FL model performance decreases when quantity imbalance is introduced. The quantity per client hinders their ability to develop a robust global model that effectively learns from various languages.

For a more in-depth investigation of the impact of the aggregation algorithm employed during the non-IID FL process to solve the performance gap, we experimented with more than one aggregation algorithm, namely FedProx and FedAvgM. FedProx differs from FedAvg in the loss function used locally at each client, considering the difference between the last global and current local models. FedAvgM differs from FedAvg in the global updating phase as it applies momentum on the server side. Nevertheless, their performances were comparable, and no specific aggregation algorithm was particularly notable. Using FedAvg as an example, the local models’ parameters are combined by means of a weighted sum to form the global model’s parameters, with the weights being directly proportional to the quantity of training data for each client. Consequently, the presence of non-IID data can have a negative impact on the accuracy of the FedAvg algorithm. Since the distribution of each local dataset is highly different from the global distribution, the local objective of each client is inconsistent with the global optima. This can interpret the low accuracy achieved for the English and Korean languages in quantity imbalance settings of Table 2 for FedAvg, since they had a one-tenth share of the total data. This is observed when there is a skewed distribution across different languages/clients, coupled with a potential imbalance in the distribution among various classes. In these specific languages, FedProx and FedAvgM perform slightly better than FedAvg. Both algorithms extend the conventional FedAvg algorithm by integrating regularizers to ensure that the parameters’ updates do not deviate excessively from the parameters established in the previous communication round. However, similar to FedAvg, they do not transfer knowledge among different clients. Compared to published literature, similar findings were observed in Gamal et al.,^24^ where FL was applied for a multilingual emoji prediction task. The non-IID results also showed a considerable decline in the model’s performance, specifically when the uneven distribution of data was introduced to clients. Another very recent study^15^ also aimed at detecting depression in only two languages, English and Chinese, using text sourced from Reddit, X (formerly Twitter), and Weibo. The datasets used ranged in size from 5,000 to 7,000 records; hence, the data for each client were relatively balanced. While the reported results do not show the performance per language, their findings show that FedProx and FedAvgM show slightly better improvement when compared to FedAvg and that FedAPFL^25^ (adaptive personalized federated learning) had the best AUC performance.

### Limitations of the study

The objective of this study was to examine the process of transferring knowledge between various clients in a multilingual FL setting for the mental health domain. However, our findings indicate that the utilization of similar-sized datasets across clients, even in diverse languages, enables the generalization of the FL architecture to identify depression through X posts. Furthermore, this approach can be extended to encompass patient notes or electronic health records, offering the added benefit of safeguarding patient privacy by avoiding data exchange between local institutions through implementing an FL strategy. However, there is still a limitation in existing FL techniques, as they still fall short on imbalanced non-IID data. The existing aggregation methods cannot handle the non-IID heterogeneity challenges observed in multilingual classification tasks, and the dissemination of knowledge among various clients is not achieved. Future research should explore more sophisticated and task-specific aggregation methods that consider each client’s contributions. Other techniques that help mitigate these challenges include knowledge distillation, which requires a shared auxiliary dataset.^26^^,^^27^

Another limitation is the lack of a unified standard for collecting or annotating depression detection datasets. Except for the English and Spanish datasets, which identified a depressed user in a systematic approach before collecting their social posts, the datasets employed a keyword-based approach for data gathering. The reliance on keyword-based approaches is a notable constraint because it causes the training process to focus predominantly on identifying depressive patterns based on specific words, making the fine-tuning of the LM somewhat too specific. We acknowledge that this limitation is not unique to our work but is a broader challenge impacting depression detection research, including local and centralized methodologies. However, it is important to note that the data collection strategy significantly contributes to the high performance observed in local training. When fine-tuning a multilingual model on a single language, the results are often comparable, and sometimes superior, to those achieved with centralized learning and FL. This is mainly because monolingual fine-tuning allows the model to adapt more effectively to the particular language’s unique linguistic characteristics and depression-related keywords, leading to improved performance in detecting depressive patterns. Additionally, multilingual LMs’ proficiency in handling diverse linguistic contexts and idioms across different languages offers a more advantageous approach to depression detection than other deep learning techniques. However, including unrelated languages in multilingual fine-tuning might not be beneficial but could result in negative interference due to conflicting gradients.^28^^,^^29^

### Conclusion and future work

This work investigates the use of multilingual FL in mental health by focusing on the challenge of detecting depression from social media posts. Our research covers English, Arabic, Russian, Spanish, and Korean languages, analyzing a total of 24,000 posts. The state-of-the-art multilingual BERT-based model was utilized to analyze the input text and categorize social media posts into either depression or non-depression classes. We contributed an in-depth comparative analysis of systematic multicentric configurations, encompassing a monolingual setting that partitioned data by language and underwent local training, a crosslingual setting that merged data and underwent training on a central server, and FL settings that distributed data among different clients in either IID or non-IID setups. Our analysis has provided insights into the effectiveness of FL algorithms in relation to LMs, text quantity variations, and class-label imbalances for extracting knowledge from multilingual patient-authored text data. The outcomes of our study align with our initial assumptions that FL in IID setups showed comparable performance to both the centralized and local models. However, due to the nature of the multilingual data, the datasets are most accurately represented using non-independent and non-identically distributed settings. Our work aimed to simulate a realistic scenario in which each dataset is associated with a single entity, ensuring that the data remain independent. The results of our study emphasize the difficulties in modifying and improving FL for datasets that contain many languages. Specifically, it points to the necessity for enhanced aggregation schemes among clients. Although our study provides an in-depth investigation into these domains, it emphasizes the necessity for further research that improves and broadens the usefulness of FL in analyzing patient data from several languages and cultures. We present initial findings demonstrating the potential of the FL paradigm in facilitating global collaboration among institutions to develop machine learning models that serve the mental health domain. One promising area for further investigation is the impact of language relatedness and the grouping of languages into different families. These families often share grammatical structures, vocabulary, etymologies, and writing systems. We aim to explore the FL approach for related languages or the same language expressed through different dialects. The Arabic language, with its diversity of dialects, is a prime example of such exploration. Another future perspective is exploring the scalability of FL, envisioning a model where patients themselves could serve as clients in the learning process. This expansion has significant implications for personalized healthcare, potentially enabling more inclusive and diverse data analysis across languages.

## Experimental procedures

### Resource availability

#### Lead contact

Further information and requests for resources and reagents should be directed to and will be fulfilled by the lead contact, Samar Samir Khalil (s.s.khalil@liacs.leidenuniv.nl).

#### Materials availability

This study did not generate new data.

#### Data and code availability

For the depression classification task, the authors did not collect new data but used existing, previously collected datasets. Three out of the five datasets are publicly available and can be downloaded directly: Arabic,^30^ Russian,^31^ and Korean^32^ data. The English dataset was obtained by following the instructions found online,^33^ and the Spanish data were obtained through personal communication with the corresponding author of Leis et al.^34^ The research proposal adheres to the ethics principles of Leiden University and was approved by the university’s ethics review committee. The code used for the training, testing, and pre-processing-related scripts can be accessed at the Zenodo repository.^35^ Any additional information required or questions regarding the data or code are available from the lead contact upon request.

### Data

As individuals increasingly utilize platforms like Facebook, X (formerly Twitter), and Reddit to express their emotions and daily experiences, patterns indicative of mental well-being or distress emerge. NLP techniques can detect linguistic patterns and indicators frequently associated with depressive thoughts and actions by analyzing vocabulary choices, sentiment expressed, and subtle linguistic nuances.^36^ This paper uses five textual datasets on depression from social media platforms. It is worth noting that some of the datasets utilized in our experiments are relatively limited in size. Nevertheless, this aligns with the practical scenarios of FL, as individual clients typically possess varying restricted amounts of data. The following datasets from five languages are utilized.

#### English

CLPsych 2015 Shared Task^37^ data contain anonymized X timelines of 1,988 users associated with depression or post-traumatic stress disorder or control users with an average of 2,267 posts per user.

#### Arabic

The Arabic_tweets_10000 dataset includes X posts that reflect depression in Modern Standard Arabic, Egyptian, and Gulf dialects. Data were obtained by using a combination of words that express mental illness, such as depression proclivity. Posts were cleaned, checked for duplication, and manually annotated by Nassar et al.^30^

#### Russian

Narynov et al.^38^ collected a dataset from public accounts of the VKontakte social network by querying its API with keywords suggested by psychologists to indicate depression. VKontakte is one of the most used social media platforms by the Commonwealth of Independent States youth, whose native language is Russian.

#### Spanish

Leis et al.^34^ followed a two-step process to collect depression data from X. First, they identified timelines of 90 users who publicly stated on their X profile that they suffer from depression. Next, they gathered depressive posts by manually selecting posts from the timelines of previously identified users. Similarly, 450 users were considered as the control group for collecting non-depressive posts.

#### Korean

Similarly, Cha et al.^39^ identified Korean-speaking users on X and collected up to 100 recent postings for thousands of Korean users. The authors used Lexicon-based labeling to annotate the collected posts and published a subset of the collected data. We used the publicly available subset of the X data.

Table 5 summarizes the sources, sizes, and examples of the datasets utilized for each class. The data were initially provided for both the English and Spanish datasets at the user level. To adapt these data for our post-level classification task, we collected the publicly available 1,000 depressed Spanish posts provided by data owners and balanced them with 1,000 non-depressed posts. These non-depressed posts were randomly selected from the control group of users. The English CLPsych 2015 Shared Task data were completely not annotated. Therefore, we randomly selected posts from users identified as depressed. The first author annotated each post as either depressed or non-depressed. This process continued until 550 posts were labeled as depressed. To ensure the reliability of these labels, the second author also annotated the same 550 posts. We then selected 500 posts that had consistent annotations from both authors. We followed the same approach as with the Spanish data for the non-depressed posts, ensuring balanced and well-annotated post-level data for our analysis.

**Table 5:** Summary for the five used datasets with examples from each class

Language	Source	Datasets size	Example
English	X	1,000	Please don’t worry about me. I’ll be okay. You won’t make me feel worse.^a^
English	X	1,000	I need to wash my clothes before I can pack.^a^
Arabic	X	10,000	الواحد بيجاهد عشان ميدخلش ف اكتئاب واله
Arabic	X	10,000	زوجة سعيدة حياة سعيد
Russian	VKontakte	10,000	Здравствуйте Я пришла вызывать к себе жалость
Russian	VKontakte	10,000	Айдын зато благодаря такому пафосуесть тема для обсуждениямаректингпиар
Spanish	X	2,000	Paciente psiquiátrico con depresión crónica
Spanish	X	2,000	Colecciono errores traducidos a tweets depresivosy an uno que otro impulso de amor
Korean	X	1,000	해피 엔딩이야말로 그 어떤 것보다 절망적인 것이다
Korean	X	1,000	작업하묜서 먹을려고 스무디 샀는데 아무맛도 안남집 앞에 카페 다신 안가

a This example is a reformulated rendition of the initial post to uphold the terms of the confidentiality agreement.

In the scope of this research, the maximum dataset size was 10,000 records, which helped reduce the trained model’s complexity. We rigorously adhered to ethical standards and ensured transparency concerning data acquisition. All datasets included in our experiments or evaluation were obtained from publicly accessible sources or acquired through direct communication with the corresponding authors of the relevant publications. When data were obtained directly from the authors, a confidentiality agreement was signed before data acquisition. This guarantees the maintenance of data integrity, the prohibition of unauthorized dissemination, and its use strictly within the bounds of our research.

### Architectures

#### LMs

For the depression detection classification task, we leveraged publicly available LMs. LMs vary in complexity, design, and number of parameters, with each model being pre-trained on different datasets to enhance their efficacy and relevance to the task. Two multilingual LMs were selected to compare their performance. The first is XLM-RoBERTa,^19^ one of the current pre-trained multilingual LMs that is prevalent and powerful. It is trained on 2.5 TB of filtered CommonCrawl data containing 100 languages with 270 M parameters for the base model. The second is TwHIN-BERT,^20^ a multilingual BERT-based post (formerly tweet) LM. It was developed at X (formerly Twitter) and trained on a dataset of 7 billion posts, which encompasses more than 100 different languages and has a total of 280 million parameters. It distinguishes itself from previous pre-trained LMs by incorporating both text-based self-supervision and a social objective derived from the many social interactions inside a TwHIN.

### Aggregation algorithms

FedAvg, the standard, most widely adopted aggregation method, is employed for FL aggregation. Two additional aggregation algorithms, FedProx^22^ and FedAvgM,^23^ are experimented and compared with the FedAvg algorithm. FedProx enhances the FedAvg algorithm by making adjustments to the local objective function. It applies l2 regularization to the difference between the global model obtained in the previous round and the current local model to compute the introduced proximal term used to update the local loss at the client. FedAvgM enhanced the FedAvg algorithm in the global updating phase by applying server momentum. Both approaches address the data heterogeneity issue and improve the model’s stability.

### Implementation details

The implemented model utilizes PyTorch^40^ as its underlying architecture. URLs, numbers, emojis, and special characters are removed from the data during the cleaning phase. The data are divided into two sets: 80% for training and 20% for testing. Within the training set, 80% is further divided into 80% for training and 20% for validation. Next, tokenization was performed to prepare the input for the LM. Additionally, we utilized the Hugging Face’s Transformers library, developed by Wolf et al.,^41^ to load the pre-trained LMs, XLM-RoBERTa and TwHIN-BERT. We fine-tuned the LMs for 10 epochs. The AdamW optimizer^42^^,^^43^ was used during the fine-tuning process with a learning rate of 5e−5. In our study, the Flower framework^44^ was employed for federated training and evaluation for both IID and non-IID partitioning. A total of 5 clients were assigned for FL experiments to match the number of languages included in our experiments. The FL training process consisted of 10 rounds, with each client training the model for one epoch per round. The model updated its weights after every round in the FL configurations. Due to the variance in the dataset sizes and the relatively small test size for some of the included datasets, such as English and Korean, we repeated each experiment three times, including the training and evaluation of the classification network for all four setups: local, centralized, IID, and non-IID.

### Non-IID client partitioning

**Figure 2:**
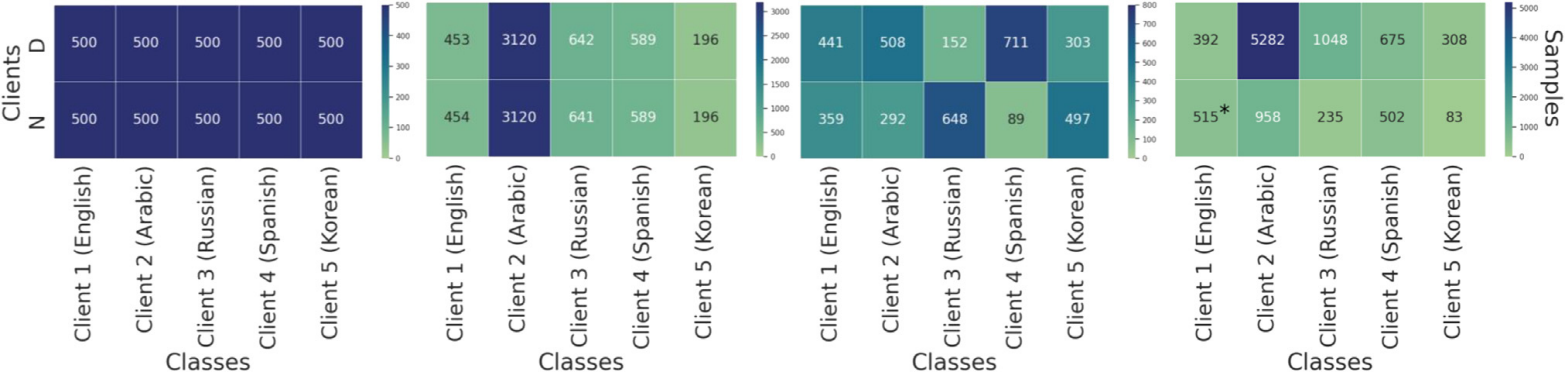
Visualization of the four non-IID setups for local dataset distribution per client. $Q_{b}L_{b}$, $Q_{b}L_{i}$, $Q_{i}L_{b}$ and $Q_{i}L_{i}$ Data are distributed using a Dirichlet distribution with β = 0.5 among five clients and two classes. The value in each rectangle is the number of data samples of a class belonging to a certain party; N denotes non-depressed, and D denotes depressed. ∗An additional 15 non-depressed English posts were added to satisfy the setup ($Q_{i}L_{i}$).

To model imbalance in non-IID multilingual data, we followed the Hsu et al.^23^ partitioning method by applying Dirichlet sampling^45^ to allocate different amounts of data samples/labels to each client. The Dirichlet distribution is commonly used to generate synthetic FL data and simulate real-world data distribution.^21^^,^^46^^,^^47^^,^^48^ A distribution $z∼Dir_{k}\left(\beta \right)$, where k is the number of clients/classes, is drawn to calculate the number of samples per clients/classes according to the formula $D_{i}=z_{i}D_{G}$, where $D_{i}$ is the number of samples per client/class and $D_{G}$ is the total number of the available samples. The degree of imbalance in the Dirichlet equation output is controlled by the parameter β. The partition is more unbalanced if β is set to a smaller value. In our experiments, we set the β value to 0.5. Four different non-IID setups were executed to explore the non-IID setting as shown in Figure 2 from left to right:

(1) Non-IID with balanced quantity and distribution-based label (non-IID:$Q_{b}L_{b}$), where the data are equally divided among clients, each with even label distribution.
(2) Non-IID with distribution-based label imbalance (non-IID:$Q_{b}L_{i}$), where the data are equally divided among clients but the label distribution follows Dirichlet sampling (β = 0.5).
(3) Non-IID with quantity imbalance (non-IID:$Q_{i}L_{b}$), where the data at each client have a different size according to Dirichlet sampling (β = 0.5) with even class-label distribution.
(4) Non-IID with quantity and distribution-based label imbalance (non-IID:$Q_{i}L_{i}$), where both the data and label distribution at each client are different according to Dirichlet sampling (β = 0.5).

In FL, classes with limited samples are referred to as tail classes.^49^ In certain cases, clients might suffer from locally imbalanced data, such as the second setup in Figure 2, where the combined dataset of all clients is globally balanced since the depressed and non-depressed classes have a total of 2,115 and 1,885 instances, respectively, across all clients. In such cases, the FL model could potentially enhance the learning process by acquiring knowledge of tail classes through interactions with other clients. On the other hand, the amounts of data samples of different labels in each client are different in the last setup in Figure 2. For example, client 2 has 5,282 samples of the depressed class and only 958 samples of the non-depressed class. It is worth noting that in the ($Q_{i}L_{i}$) setup, an additional 15 English non-depressed posts were added from the control user group to meet the requirements of the experimental design.
